# The in vitro cross-reactivity and blood coagulation potential of recombinant porcine factor VIII in Japanese patients with acquired hemophilia A

**DOI:** 10.1007/s12185-024-03854-5

**Published:** 2024-10-30

**Authors:** Masahiro Takeyama, Kana Sasai, Yasuo Miyaguchi, Kenichi Ogiwara, Shoko Furukawa, Naruto Shimonishi, Yuto Nakajima, Hitoshi Ueda, Keiji Nogami

**Affiliations:** 1 Department of Pediatrics, Nara Medical University, 840 Shijo-cho, Kashihara, Nara 634-8522 Japan; 2 Takeda Pharmaceutical Co., Ltd., Tokyo, Japan; 3 The Course of Thrombosis and Hemostasis Molecular Pathology, Nara Medical University, Kashihara, Nara Japan; 4 Advanced Medical Science of Thrombosis and Hemostasis, Nara Medical University, Nara, Japan

**Keywords:** Acquired hemophilia A, Autoantibodies, Factor VIII, Hemostasis, Japan

## Abstract

Recombinant porcine factor VIII (rpFVIII) is a hemostatic agent for acquired hemophilia A (AHA). Cross-reaction of auto-antibodies against rpFVIII has been reported, although no data are available in Japanese patients. This study investigated the cross-reactivity and coagulation potential of rpFVIII in plasma samples from Japanese patients with AHA. Cross-reactivity was calculated as the ratio of anti-porcine FVIII inhibitor titer (pFVIII-INH) to human FVIII inhibitor titer. Comprehensive coagulation potential was assessed by clot waveform analysis (CWA) and thrombin generation assay (TGA) in samples spiked with rpFVIII (equivalent to 200 U/kg). Nine of 16 plasma samples (56.3%) had positive pFVIII-INH, with a median cross-reactivity of 1.2%. FVIII activity (FVIII:C) was restored to > 100% in all samples upon spiking with rpFVIII, but was weakly correlated with pFVIII-INH. CWA parameters and most TGA parameters were restored to normal upon spiking with rpFVIII; correlation of these parameters with FVIII:C was similar to that observed in controls. Overall, cross-reactivity to rpFVIII in Japanese patients was similar to that reported in Caucasian patients. Our results suggest that an initial clinical dose of 200 U/kg rpFVIII could restore coagulation potential to normal, and that FVIII:C monitoring after rpFVIII administration may be more informative than pFVIII-INH before administration.

**Supplementary Information:**

The online version contains supplementary material available at 10.1007/s12185-024-03854-5.

## Introduction

Acquired hemophilia A (AHA), unlike congenital hemophilia A (CHA), is an autoimmune disease characterized by a bleeding tendency. AHA is caused by the development of autoantibodies against coagulation factor VIII (FVIII), which leads to inhibition of FVIII function [1]. Patients with AHA (PwAHA) typically present with extensive subcutaneous and muscle hemorrhages, but can also occasionally develop gastrointestinal hemorrhage and, rarely, intracranial hemorrhage, which can be fatal [1]. Treatment of PwAHA includes hemostatic therapy for hemorrhages and immunosuppressive therapy to suppress autoantibody production [2]. Bypassing agents are available for hemostatic therapy, and three such agents are clinically available in Japan as of 2022: recombinant activated factor VII (rFVIIa), plasma-derived activated prothrombin complex concentrate (aPCC), and plasma-derived activated factor VII/factor X complex concentrate (FVIIa/FX) [3]. Furthermore, a bispecific monoclonal antibody against activated factor IX (FIXa)/FX (emicizumab) was approved as a hemostatic treatment for PwAHA in Japan in June 2022 [4].

Porcine FVIII (pFVIII) has been shown to function as an alternative to human FVIII (hFVIII) in human plasma, by binding to FIXa and FX, while also being sufficiently different in structure to hFVIII, rendering it less susceptible to inactivation by circulating anti-hFVIII inhibitory antibodies [5]. An advantage of pFVIII over bypassing agents and emicizumab is that it enables treatment monitoring through measurement of FVIII activity (FVIII:C), which can be used as an indicator for reliable hemostasis. In the USA and Europe, plasma-derived pFVIII was originally used clinically for patients with hemophilia A who have FVIII inhibitors [6]; however, it was withdrawn from the market owing to concerns about porcine parvovirus contamination in the porcine plasma [5, 7]. ln Japan, the safety and efficacy of plasma-derived pFVIII were demonstrated in a multicenter clinical trial [8]; however, it was not used clinically owing to the same concerns regarding porcine parvovirus [5, 7, 8]. Therefore, a recombinant pFVIII (rpFVIII) product was developed and approved to address the risk of parvovirus infection and is now available clinically in the USA and Europe [9]. In Japan, rpFVIII was approved for treating bleeding episodes in adults with AHA in 2024 [10].

For rpFVIII to function as an alternative to hFVIII in plasma, a sufficient similarity in amino acid sequence is required between rpFVIII and hFVIII. Therefore, when rpFVIII is administered to PwAHA, it is inevitable that the anti-hFVIII autoantibodies in PwAHA may cross-react to and reduce the efficacy of rpFVIII. The cross-reactivity rate (pFVIII inhibitor titer in Bethesda units [BU]/hFVIII inhibitor titer in BU) was reported as a mean (standard deviation [SD]) of 8.7% (27.8%) in a clinical study in PwAHA [11]. An in vitro investigation of plasma samples from PwAHA in the GTH‐AH01/2010 study found a median cross-reactivity rate of 0% (interquartile range [IQR]: 0–3.2%) [12]. Although this global study also reported that cross-reactive pFVIII inhibitors reduced the FVIII:C of rpFVIII, such findings have not been verified or replicated in a Japanese context. It is, therefore, crucial to investigate the cross-reactivity of hFVIII inhibitors in Japanese PwAHA, to inform appropriate use of rpFVIII in clinical practice.

Comprehensive coagulation assays such as thrombin generation assay (TGA), clot waveform analysis (CWA), and rotational thromboelastometry are used for hemostatic monitoring of bypassing agents and emicizumab in patients with CHA with inhibitors (PwCHA-INH) [13–16]. After spiking rpFVIII into plasma from PwCHA-INH, pFVIII inhibitor titer was demonstrated to be inversely correlated with parameters such as endogenous thrombin potential and peak height in TGA [17]. Although a large amount of data has been reported regarding comprehensive blood coagulation potential in PwCHA-INH, such data are limited in PwAHA. Notably, no data on the use of comprehensive coagulation assays for monitoring rpFVIII have been reported in PwAHA. Thus, this study was performed to investigate the cross-reactivity of anti-hFVIII autoantibodies against rpFVIII in Japanese PwAHA. In addition, as supporting data for the cross-reactivity analysis, the blood coagulation potential after spiking rpFVIII into plasma from PwAHA was assessed by the measurement of FVIII:C, TGA, and CWA.

## Materials and methods

### Reagents

FVIII-deficient plasma derived from PwCHA-INH (George King Bio-Medical, Inc., Overland Park, KS, USA), activated partial thromboplastin time (aPTT) reagent (Revohem APTT-SLA, Sysmex Corp., Kobe, Japan), and recombinant human tissue factor (Innovin, Dade, Marburg, Germany) were purchased from the indicated vendors. Phospholipid vesicles containing 10% phosphatidylserine, 60% phosphatidylcholine, and 30% phosphatidylethanolamine (Sigma-Aldrich, St. Louis, MO, USA) were prepared as previously described [18].

### Patients and samples

Previously collected and stored plasma samples from Japanese PwAHA were used for this study. Patients had agreed to future use of their plasma samples by Nara Medical University, and measurements for this study were performed after website opt-out in compliance with the Japanese Ethical Guidelines. Plasma samples were obtained between January 2011 and April 2022 and stored at below − 80 °C until the assay measurements. This study was approved by the Medical Research Ethics Committee of Nara Medical University.

### Preparations of plasma test samples

Patient plasma spiked with rpFVIII at a dose equivalent to 200 U/kg (concentration: 5 U/mL) was used in each assay. The relationship between circulating plasma volume and body weight was calculated from the equation as follows: circulating plasma volume = body weight × 1000 × 0.08 × 1/2 (assuming a hematocrit of 50%). Control samples were prepared by mixing FVIII-deficient plasma with rpFVIII at concentrations of 0, 0.3, 0.625, 1.25, 2.5, and 5.0 U/mL, resulting in equivalent rpFVIII concentrations of 0, 12, 25, 50, 100, and 200 U/kg, respectively.

### Inhibitor epitope mapping

Major epitopes of the inhibitor antibodies were determined by sodium dodecyl–sulfate polyacrylamide gel electrophoresis and Western blotting using recombinant FVIII and thrombin-cleaved recombinant FVIII [19].

### FVIII inhibitor measurement

Anti-hFVIII inhibitor titers were measured using the Bethesda method [20]. Regarding anti-pFVIII inhibition, dilution series of patient plasma with imidazole buffer (0.1 M NaCl with 0.05 M imidazole, pH 7.4) containing 0.25% human serum albumin were prepared and mixed with an equivalent volume of FVIII-deficient plasma, to which rpFVIII (Takeda Pharmaceuticals, Tokyo, Japan) was added at a concentration of 1 U/mL. After incubation at 37 °C for 2 h, the residual FVIII:C was measured by a one-stage clotting assay. Using a mixture of imidazole buffer and FVIII-deficient plasma with rpFVIII as the control, the same assay was performed. The residual FVIII:C relative to the control was plotted on the calibration curve and multiplied by the dilution ratio to calculate the inhibitor titer (BU/mL).

### FVIII:C measurement

In a preliminary study, FVIII:C inhibition reached a plateau 30 min after addition of hFVIII to the plasma of PwAHA. Therefore, in this study, FVIII:C was measured 30 min after rpFVIII was added. FVIII:C measurements were determined by a one-stage clotting assay, using FVIII-deficient plasma and APTT reagent on a CS-2400 automated instrument (Sysmex).

### CWA

Modified CWA was performed on the CS-2400 using an aPTT reagent as previously reported [21]. Measurements were performed 30 min after addition of rpFVIII to plasma. Clot formation was initiated by the addition of calcium chloride (20 mM). The clot waveforms obtained were computer processed using the commercial kinetic algorithm. The clot time (CT) was determined as the time to transmittance reduction to the predefined level. The rates of clotting and acceleration were computed from the first- and second-order differentials of transmittance, respectively. The minimum absolute value of the first-order differential (|min1|) represents the maximum coagulation rate, and the minimum absolute value of the second-order differential (|min2|) represents the maximum acceleration of coagulation rate. Ad|min1| and Ad|min2| were defined as the maximum coagulation velocity obtained from the first and second derivative of CWA, based on the clot waveform in which the transmittance of post-coagulation phase was adjusted to the 0% level, to account for maximal elimination of the influence of plasma fibrinogen concentration [22].

### TGA

Calibrated automated TGA was performed as previously described [23, 24]. Plasma samples were pre-incubated for 30 min with rpFVIII (200 U/kg; 80 μL) and 20 μL of trigger reagent containing phospholipids and recombinant human tissue factor (final concentrations: 4 μM and 1 pM, respectively). Measurements were then recorded after the addition of 20 μL reagent containing calcium chloride and fluorogenic substrate (final concentrations: 16.7 and 2.5 mM, respectively). The development of fluorescent signals was monitored using a Fluoroskan Ascent microplate reader (Thermo Fisher Scientific). Data analyses were performed using the manufacturer’s software to derive the standard parameters; lag time, time to peak, peak thrombin, and endogenous thrombin potential.

### Statistical analysis

Cross-reaction was considered positive if the pFVIII inhibitor titer was at least 0.6 BU/mL. The cross-reactivity was calculated by dividing the pFVIII inhibitor titer by the hFVIII inhibitor titer. The Pearson correlation coefficient was used to analyze the relationship between pFVIII inhibitor titers and hFVIII inhibitor, FVIII:C, CWA, and TGA parameters. A Dunnett’s multiple comparison test was used to compare the differences in TGA and CWA parameters between plasma from PwAHA (before and 30 min after spiking rpFVIII to plasma) and normal plasma. *p* values below 0.05 were considered statistically significant.

## Results

### Patient characteristics, FVIII:C, and FVIII inhibitor titers

In total, 16 plasma samples from PwAHA were used for this study (Table 1). Mean (SD) patient age was 69.3 (15.0) years (Table 2). Three of the 16 patients (19%) had concurrent malignancies, one patient (6%) had an autoimmune disease, and one (6%) was post-partum. The median (range) FVIII:C and hFVIII inhibitor titers before addition of rpFVIII were 0.6% (0–33%) and 85.2 (4–387) BU/mL, respectively. The specific hFVIII inhibitor epitopes could only be identified in a limited number of samples. Inhibitor epitope could not be detected in two patients (Table 1).

**Table 1:** Patient characteristics, FVIII inhibition, and cross-reactivity of recombinant porcine FVIII

Patient	Age group	Sex	Underlying condition	Inhibitor epitope (HC or LC)	FVIII:C	FVIII inhibitor (BU/mL)		Cross-reactivity	Cross-reaction	Theoretical neutralization and recovery of FVIII:C ≥ 100% by rpFVIII 200 U/kg^b^
					Pre (%)^a^	To hFVIII	To rpFVIII	pFVIII BU/hFVIII BU	pFVIII BU ≥ 0.6	
1	70s	F	Bullous pemphigoid	LC	< 0.1	126	3.43	2.7%	Yes	Yes
2	80s	F	Hypertension	n.d	< 0.1	256	2.03	0.8%	Yes	Yes
3	70s	M	None	A1, A2, LC	7.5	90.0	2.98	3.3%	Yes	Yes
4	70s	M	Gastric cancer, post-operation	A2	< 0.1	134	1.40	1.0%	Yes	Yes
5	60s	M	Colon cancer, post-operation	n.d	33	156	0.49	0.3%	No	Yes
6	60s	M	Hypertension	A3C1	5.7	5.5	< 0.30	0.0%	No	Yes
7	70s	M	Prostatomegaly	C2	2.1	387	19.9	5.1%	Yes	No
8	70s	M	Gastric cancer, post-operation	A2, LC	1.1	49.0	1.48	3.0%	Yes	Yes
9	70s	M	Diabetes mellitus	A2	< 0.1	274	0.99	0.4%	Yes	Yes
10	80s	F	None	A2, LC	12	4.0	< 0.30	0.0%	No	Yes
11	30s	F	Post-partum	A1, A2, LC	< 0.1	11.8	0.35	3.0%	No	Yes
12	30s	F	None	A1, A2, LC	< 0.1	20.7	< 0.30	0.0%	No	Yes
13	80s	F	Parkinson’s disease	A1, A2, LC	2.1	43.2	0.58	1.3%	No	Yes
14	50s	M	Diabetes mellitus	A2, LC, C2	1.4	104	4.05	3.9%	Yes	Yes
15	80s	F	None	LC, C2	< 1.0	12.9	0.4	3.1%	No	Yes
16	60s	M	Hypertension	A1, A3C1	< 1.0	80.3	0.7	0.9%	Yes	Yes
Mean ± SD (median)					4.1 ± 8.4 (0.6)	109.7 ± 111.5 (85.2)	2.4 ± 4.8 (0.8)	1.8 ± 1.6 (1.2)		

*BU* Bethesda units, *F* female, *FVIII* factor VIII, *FVIII:C* factor VIII activity, *HC* heavy chain (A1 and A2 domains) , *hFVIII* human FVIII, *LC* light chain (A3, C1 and C2 domains) , *M* male, *n.d.* not determined, *pFVIII* porcine FVIII, *rpFVIII* recombinant porcine FVIII, *SD* standard deviation

^a^FVIII activity measured before spiking with rpFVIII

^b^Based on Japanese Society on Thrombosis and Hemostasis guideline

**Table 2:** Summary of FVIII inhibition and cross-reactivity analysis

	Patients (*N* = 16)
Age (years)	
Mean ± SD	69.3 ± 15.0
Median	73
Q1, Q3	65.0, 80.0
Min, max	36, 87
Sex (*n*, %)	
Male	9 (56.3)
Female	7 (43.8)
Underlying condition (*n*, %)	
Malignancy	3 (18.8)
Autoimmune disease	1 (6.3)
Infection	0 (0.0)
Drug	0 (0.0)
Pregnancy	1 (6.3)
Other	7 (43.8)
Idiopathic	4 (25.0)
FVIII:C pre-spiking with rpFVIII (%)	
Mean ± SD	4.1 ± 8.4
Median	0.6
Q1, Q3	0.0, 3.9
Min, max	0, 33
FVIII inhibitor titer	
Anti-hFVIII (BU/mL)	
Mean ± SD	109.7 ± 111.5
Median	85.2
Q1, Q3	16.8, 145.0
Min, Max	4, 387
Anti-pFVIII (BU/mL)	
Mean ± SD	2.4 ± 4.8
Median	0.8
Q1, Q3	0.4, 2.5
Min, max	0, 20
Cross-reactivity (pFVIII BU/mL/hFVIII BU/mL) (%)	
Mean ± SD	1.8 ± 1.6
Median	1.2
Q1, Q3	0.3, 3.1
Min, max	0.0, 5.1
Cross-reaction (pFVIII BU ≥ 0.6) (*n*, %)	
No	7 (43.8)
Yes	9 (56.3)
Theoretical neutralization and recovery of FVIII:C to over 100% by 200 U/kg rpFVIII (*n*, %)^a^	
No	1 (6.3)
Yes	15 (93.8)

*BU* Bethesda units, *FVIII* factor VIII, *FVIII:C* factor VIII activity, *hFVIII* human factor VIII, *pFVIII* porcine factor VIII, *rpFVIII* recombinant porcine factor VIII, *SD* standard deviation

^a^Based on Japanese Society on Thrombosis and Hemostasis guidelines

### Cross-reactivity to rpFVIII in plasma from PwAHA

**Fig. 1:**
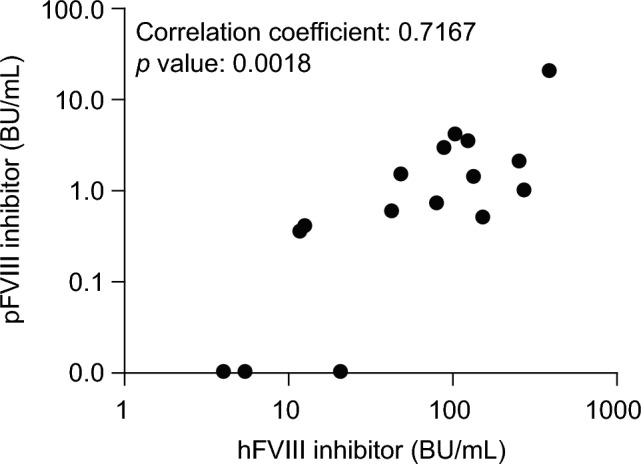
Correlation between hFVIII inhibitor titer and pFVIII inhibitor titer in plasma from PwAHA (*N* = 16). Anti-hFVIII inhibitor titers were measured using the Bethesda method. For the anti-pFVIII inhibitor, dilution series of plasma samples were mixed with FVIII-deficient plasma, to which rpFVIII (1 U/mL) was added, followed by measurement of FVIII:C and calculation of anti-pFVIII inhibitor titer as described in the Methods. The Pearson correlation coefficient was used to analyze the relationship between pFVIII inhibitor titer and hFVIII inhibitor titer. *p* < 0.05 was considered statistically significant. *BU* Bethesda units, *FVIII* factor VIII, *FVIII:C* factor VIII activity, *hFVIII* human factor VIII, *pFVIII* porcine factor VIII, *PwAHA* patients with acquired hemophilia A, *rpFVIII* recombinant porcine factor VIII

The median (range) pFVIII inhibitor titer was 0.8 (0–20) BU/mL and the median (range) cross-reactivity (pFVIII inhibitor titer/hFVIII inhibitor titer) was 1.2% (0–5.1%) (Table 2). The pFVIII inhibitor titer was at least 0.6 BU/mL in nine samples (56%). The mean cross-reactivity was 1.2% in samples that had the inhibitor epitope only on the hFVIII heavy chain, 2.7% in samples that had the epitope only on the light chain, and 1.9% in samples that had the epitope on both the heavy and light chains, indicative of no clear effect of epitope location on cross-reactivity. The proportion of samples that could theoretically neutralize pFVIII inhibitors and increase plasma FVIII:C to at least 100% (assuming a recovery rate of 2.0) when treated with an initial clinical dose of 200 U/kg rpFVIII was 15 out of 16 (94%), when calculated according to the Japanese Society on Thrombosis and Hemostasis clinical practice guidelines for hemostatic treatment of congenital hemophilia with inhibitors [25]. There was a strong positive correlation between the hFVIII inhibitor titer and the pFVIII inhibitor titer (Pearson coefficient: 0.7167). All samples with hFVIII inhibitor titers of at least 49 BU/mL had pFVIII inhibitor titers of at least 0.6 BU/mL (Fig. 1).

### FVIII:C in plasma from PwAHA after spiking with rpFVIII

**Fig. 2:**
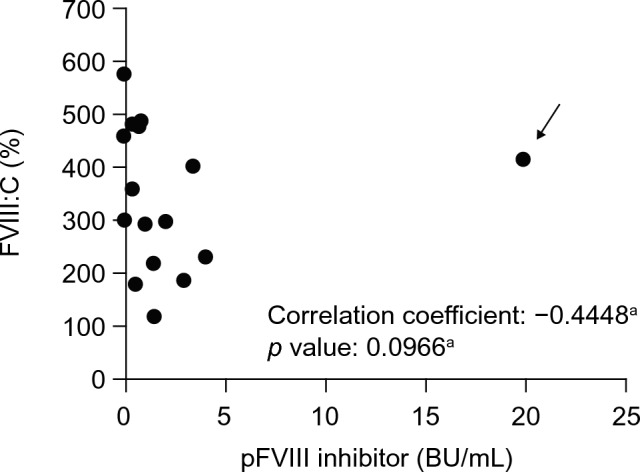
Correlation between pFVIII inhibitor titer and FVIII:C in plasma from PwAHA (*N* = 16). Anti-pFVIII inhibitor titers were measured and calculated as described in the Methods. The Pearson correlation coefficient was used to analyze the relationship between pFVIII inhibitor titers and FVIII:C. *p* < 0.05 was considered statistically significant. *BU* Bethesda units, *FVIII:C* FVIII activity, *pFVIII* porcine factor VIII, *PwAHA* patients with acquired hemophilia A. ^a^The data indicated by the arrow were considered as an exceptionally high titer (19.9 BU/mL) and was excluded from the calculation. Therefore, the statistical result shown was based on the remaining 15 samples

Thirty minutes after spiking plasma samples from PwAHA with 200 U/kg of rpFVIII (equivalent to 5 U/mL), the median FVIII:C was 327% (range: 116–573%), with all samples having FVIII:C levels over 100% (Fig. 2). Although a negative correlation between pFVIII inhibitor and FVIII:C was expected after spiking with rpFVIII, unexpectedly the Pearson coefficient was 0.0238, suggesting little correlation. However, when one sample with an exceptionally high pFVIII inhibitor titer of 19.9 BU/mL was excluded from the calculation, the Pearson coefficient was − 0.4448, suggesting the tendency to a weak negative correlation without statistical significance (*p* = 0.0966).

### Comprehensive coagulation potentials of plasma from PwAHA after spiking with rpFVIII—CWA-based assessment

**Fig. 3:**
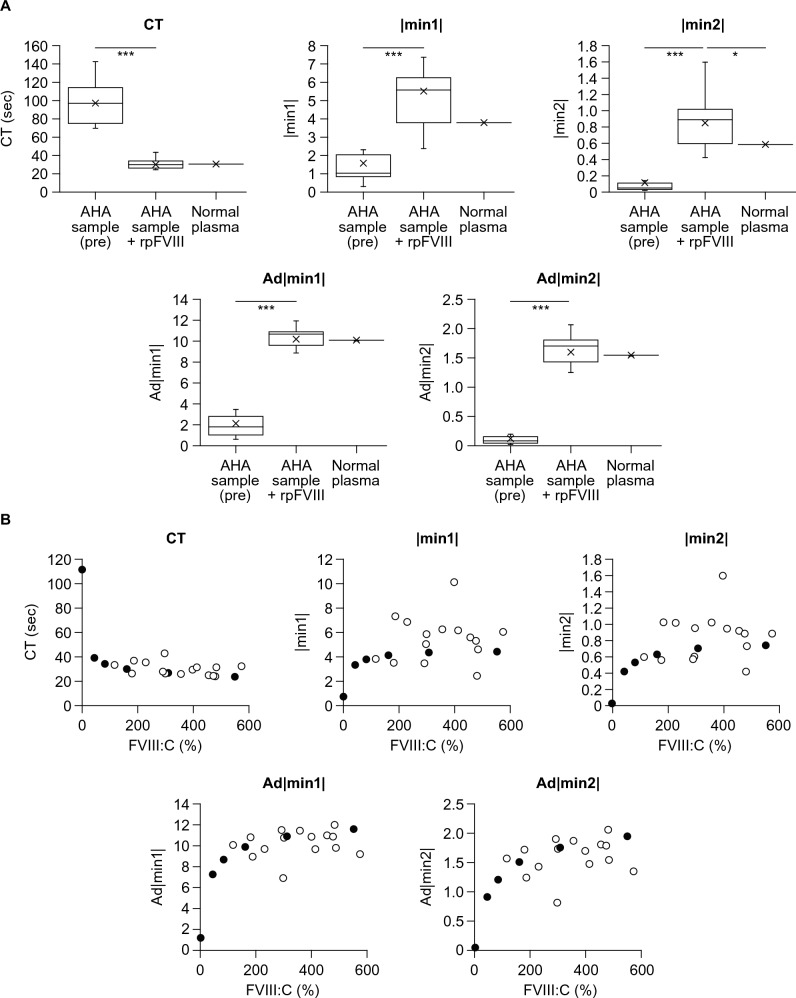
Coagulation potentials measured by CWA in plasma samples from PwAHA after spiking with rpFVIII. **A** aPTT-based CWA measurements were performed as described in the Methods for three groups: patient plasma pre-spiking with rpFVIII, patient plasma incubated with rpFVIII (5 U/mL; equivalent to 200 U/kg) for 30 min, and pooled normal plasma. A Dunnett’s multiple comparison test was used to compare the differences in parameters between the three groups. *p* < 0.05 was considered statistically significant. *p < 0.05, ***p < 0.0001. **B** Patient plasma incubated with rpFVIII (5 U/mL; equivalent to 200 U/kg) for 30 min (open circle) and FVIII-deficient plasma mixed with rpFVIII (0, 0.3, 0.625, 1.25, 2.5, and 5.0 U/mL as an equivalent dose to 12, 25, 50, 100, and 200 U/kg, respectively) and control plasma (closed circle) were assessed by aPTT-based CWA as described in the Methods. *Ad|min1|* adjusted |min1|, *Ad|min2|* adjusted |min2|, *aPTT* activated partial thromboplastin time, *CT* clotting time, *CWA* clot waveform analysis, *FVIII* factor VIII, *FVIII:C* FVIII activity, *|min1|* maximum coagulation velocity, *|min2|* maximum coagulation acceleration, *PwAHA* patients with acquired hemophilia A, *rpFVIII* recombinant porcine factor VIII

Compared with before the addition of rpFVIII, all clot waveform parameters (CT, |min1|, |min2|, Ad|min1|, and Ad|min2|) were restored to normal levels after spiking with rpFVIII (Fig. 3A). The |min2| was restored to significantly higher levels than normal plasma after addition of rpFVIII. Overall, the relationships between FVIII:C and clot waveform parameters after rpFVIII addition were similar to those observed in control samples (Fig. 3B). When the FVIII:C was high after spiking with rpFVIII, Ad|min1| and Ad|min2| tended to be restored to a greater extent. For CT, |min1|, and |min2|, there was less association with FVIII:C, which was also the case for the control sample. Some samples showed lower or higher values than the control samples. The sample that had low values for |min1| and |min2| (2.43 and 0.42, respectively) after spiking with rpFVIII (patient 11) also had slightly low peak thrombin in TGA. Conversely, the sample that had high values for |min1| and |min2| (10.1 and 1.59, respectively) after spiking (patient 1) had high peak thrombin in TGA (503 nM) (Supplementary Tables S1, S2). However, no correlation with pFVIII inhibitor titer was observed for all parameters (Supplementary Fig. S1).

### Comprehensive coagulation potentials of plasma from PwAHA after spiking with rpFVIII—TGA-based assessment

**Fig. 4:**
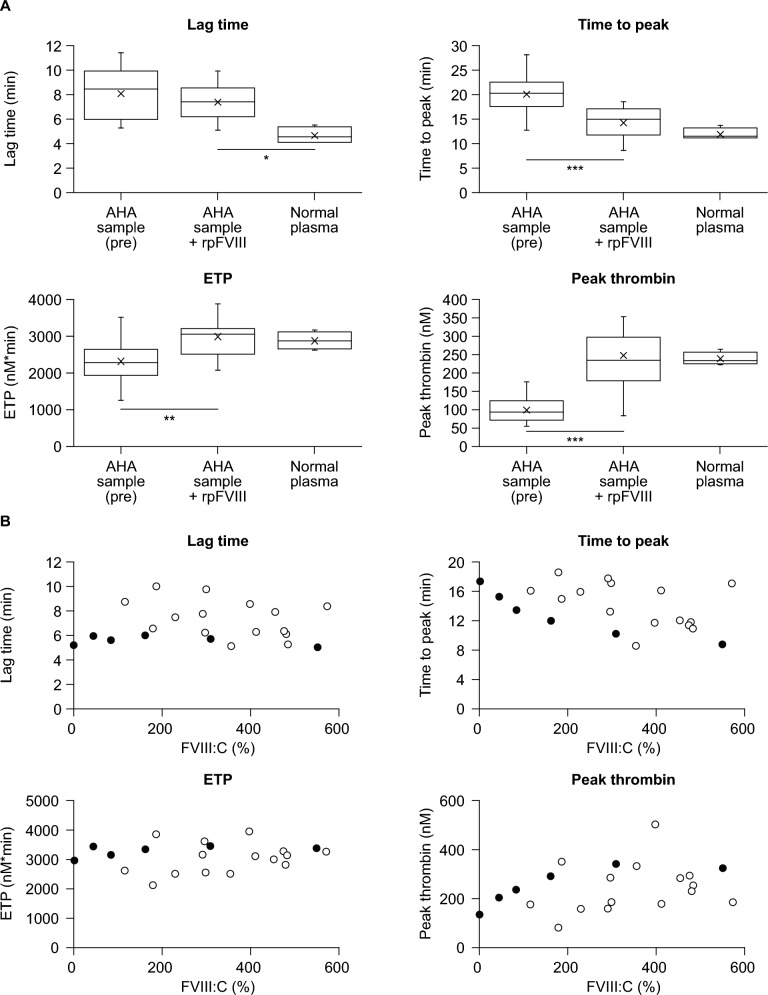
Coagulation potentials measured by TGA in plasma samples from PwAHA after spiking with rpFVIII. **A** TF-triggered TGA measurements were performed as described in the Methods for three groups: patient plasma pre-spiking with rpFVIII, patient plasma incubated with rpFVIII (5 U/mL; equivalent to 200 U/kg) for 30 min, and pooled normal plasma. A Dunnett’s multiple comparison test was used to compare the differences in parameters between the three groups. *p* < 0.05 was considered statistically significant. *p < 0.05, **p < 0.005, ***p < 0.0001. **B** Patient plasma incubated with rpFVIII (5 U/mL; equivalent to 200 U/kg) for 30 min (open circle), and FVIII-deficient plasma mixed with rpFVIII (0, 0.3, 0.625, 1.25, 2.5, and 5.0 U/mL as an equivalent dose of 12, 25, 50, 100, and 200 U/kg, respectively) and control plasma (closed circle) were assessed by TF-triggered TGA as described in the Methods. *AHA* acquired hemophilia A, *FVIII* factor VIII, *FVIII:C* FVIII activity, *ETP* endogenous thrombin potential, *PwAHA* patients with acquired hemophilia A, *rpFVIII* recombinant porcine factor VIII, *TF* tissue factor, *TGA* thrombin generation assay

After addition of rpFVIII, all TGA parameters except lag time were restored to levels comparable to those of normal plasma (Fig. 4A). Overall, time to peak and peak thrombin tended to be restored as FVIII:C increased, but some samples showed low or high values regardless of FVIII:C (Fig. 4B). In one sample (patient 5), the FVIII:C was 179%, but the peak thrombin was low (80.67 nM) (Supplementary Table S2). The CWA |min1| and |min2| values for this sample were 3.49 and 0.56, respectively (Supplementary Table S1), similar to those in the control sample (Fig. 4B). There was no correlation with pFVIII inhibitor titer for any of the TGA parameters (Supplementary Fig. S2).

## Discussion

Since 2014, rpFVIII has been approved and used in clinical practice in the USA and Europe, and is positioned as a first-line hemostatic treatment for PwAHA alongside bypassing agents. One of the challenges of using rpFVIII products is the cross-reaction of anti-hFVIII autoantibodies in PwAHA. Analyses of the GTH-AH 01/2010 study showed that 44% of patient plasma samples cross-reacted to rpFVIII [12]. The results obtained in our study showed that rpFVIII elicited a cross-reaction in 56% of plasma samples from PwAHA, similar to the GTH-AH 01/2010 data. In addition, our study showed a median cross-reactivity of 1.2% (IQR 0.3–3.1%, range 0–5.1%), similar to the cross-reactivity reported in the GTH-AH 01/2010 study (0%; IQR 0–3.2%, range 0–46.7%). Together, these results indicate that the cross-reactivity of PwAHA autoantibodies to rpFVIII is similar in Japanese and Caucasian patients. In the present study, there was only one patient with a pFVIII inhibitor titer equal to or above 10 BU/mL, at 19.9 BU/mL. The inhibitors in each of the other patient samples could theoretically be neutralized with 200 U/kg of rpFVIII, resulting in increased plasma FVIII:C to at least 100%. This suggests that the initial clinical dose of rpFVIII could neutralize the cross-reactive pFVIII inhibitors found in the plasma of most PwAHA.

Data from the GTH-AH 01/2010 study suggest a tendency for greater cross-reactivity in samples containing autoantibodies against epitopes in the C1 domain [12]. In our study, cross-reactivity tended to be higher in the samples with epitopes only on the light chain (A3-C1-C2) than in the samples with epitopes only on the heavy chain (A1-A2), which is consistent with previous reports [26]. However, there were cases in which no epitope was detected, or specific epitopes were not identified. This made it difficult to verify the difference in cross-reactivity, owing to the difference in epitopes compared with the GTH-AH 01/2010 data.

FVIII:C levels exceeded 100% in all patient samples when plasma was spiked with 200 U/kg of rpFVIII (5 U/mL). Unexpectedly, FVIII:C was 412% in a sample with a pFVIII inhibitor titer of 19.9 BU/mL (patient 7). Initially, there was no correlation between FVIII:C and pFVIII inhibitor titer after addition of rpFVIII. However, upon exclusion of this sample (patient 7), a weak negative correlation without statistical significance was observed. Therefore, the pFVIII inhibitor value for patient 7 was considered an outlier. Compared with the present study, in a previous study in which the baseline pFVIII inhibitor titer ranged from less than 0.6 BU to 29 BU, there was a more pronounced negative correlation between pFVIII inhibitor titer and FVIII:C increase in patients after administration of rpFVIII [11]. However, in the present study, only one patient had a pFVIII inhibitor titer above 5 BU/mL, and this was the patient who was considered an outlier. Therefore, it is difficult to directly compare the results between the two studies. Autoantibodies in PwAHA are known to behave in a type 2 manner [19, 27], in that some endogenous FVIII:C remains even when FVIII inhibitor titer is high. Although cross-reactivity was low in our study, as indicated by the pFVIII inhibitor titer determined by the Bethesda assay, it is possible that the pFVIII inhibitor titer and FVIII:C after administration of rpFVIII behave as type 2, suggesting the influence of non-neutralizing antibodies (binding antibodies). Therefore, the recovery in FVIII:C after rpFVIII treatment is difficult to predict based on the pFVIII inhibitor titer. Rather, measuring of FVIII:C should be used for the monitoring of rpFVIII hemostasis as recommended in the previous report [11]. It should also be noted that FVIII:C was only measured at one time point (30 min) after rpFVIII addition in our study, owing to limited sample volumes. The time-dependent FVIII:C inhibitory effects of autoantibodies (e.g., at 1 or 2 h after rpFVIII addition) should be considered in future research.

Previous reports have demonstrated the utility of TGA and CWA parameters in assessing endogenous comprehensive coagulation potential in PwAHA [28]. There have been no reports evaluating TGA and CWA in PwAHA after administration of rpFVIII; therefore, our study investigated whether these assays might be useful as a monitoring index in this context. All parameters from CWA and TGA were found to restore to levels similar to normal plasma after spiking with rpFVIII. In some patient samples, the parameter levels were low. In addition, there was no correlation of these CWA and TGA parameters with pFVIII inhibitor titers overall. Previous reports have shown that peak thrombin and time to peak had a strong inverse correlation with pFVIII inhibitor titers after rpFVIII addition to the plasma from PwCHA-INH [17]. In PwCHA-INH, inhibitor behavior is known to be predominantly type 1 [19, 27], and the inhibitor titer and FVIII:C show a linear negative correlation. Because the behavior of inhibitors in PwAHA is predominantly type 2 [19, 27] and, therefore, inhibitor titers may not correlate with FVIII:C values, it is reasonable that the comprehensive coagulation potential and the pFVIII inhibitor titer did not correlate in our study. However, the overall trend of recovery was similar to that of normal plasma, suggesting that the TGA results also support the clinical efficacy of rpFVIII.

The TGA and CWA parameters tended to be restored with increasing FVIII:C, but did not tend to increase markedly even with FVIII:C of over 200%. In our study, a dose equivalent to 200 U/kg of rpFVIII (final concentration: 5 U/mL) was added, and FVIII:C exceeded 200% in many samples. However, TGA and CWA parameters associated with increased FVIII:C reached a plateau, suggesting that the levels of other coagulation factors (e.g., FX, FIX, fibrinogen) or anticoagulants (e.g., protein S, protein C, anti-thrombin) in the plasma could be rate-limiting for TGA and CWA parameters. An increase in comprehensive coagulation potential beyond normal plasma was, however, observed in some samples. In the sample from patient 1, the CWA values were high for |min1| and |min2|, but not for Ad|min1| and Ad|min2| (see Supplementary Table S1)*,* indicating the possibility that fibrinogen values may be related. The peak thrombin in TGA was also high in this sample. Considering that peak thrombin increases with increasing fibrinogen level [29], the possibility that patient fibrinogen levels were related to the high thrombin peak cannot be ruled out. In the sample from patient 5, FVIII:C increased up to 179% but TGA peak thrombin was low, while CWA |min1| and |min2| values were equivalent to control samples (see Supplementary Tables S1 and S2). In this sample, extrinsic factors (e.g., high tissue factor pathway inhibitor) rather than intrinsic factors seemed likely to be involved. Although TGA parameters recovered to levels comparable to normal plasma overall, caution may be needed because it was observed that some patients had insufficient recovery of peak thrombin when compared with the mean [SD] peak thrombin observed in control plasma (234.8 [17.7] nM). The sample from patient 11 had very low |min1| and |min2|, but only a slightly low value for peak thrombin was observed, and Ad|min1| and Ad|min2| were similar to those of the control (see Supplementary Tables S1 and S2). Therefore, the influence of low fibrinogen levels should be considered. Thus, the possibility that some patients receiving a dose of 200 U/kg of rpFVIII may have different hemostatic effects in clinical practice cannot be denied. However, the patient characteristics and factors related to this differential response failed to be identified in our study. A study involving a larger number of patients in a clinical setting would be needed to investigate this.

Overall, despite some variability in responses to rpFVIII between individual patient samples, the results of coagulation potential for most samples in this study were within the expected range, and the results largely support the efficacy and safety of rpFVIII. Given that TGA and CWA parameters could depend on the activity of coagulation factors other than FVIII, or on anticoagulants in the patient’s plasma, further investigation is still needed to determine whether TGA and CWA are appropriate monitoring tests for evaluating the hemostatic potential of rpFVIII. In our study, there were no samples in which the FVIII:C was less than 100% after spiking with 200 U/kg of rpFVIII, although this could occur in clinical use. Monitoring of FVIII:C can detect a failure to increase the coagulation potential as expected, and appropriate actions such as additional administration of rpFVIII should be taken. For the proper use of rpFVIII, monitoring of FVIII:C and clinical symptoms, and appropriate adjustment of regimens are important.

A limitation of this study was that it was not an in vivo study, but rather an in vitro study using plasma from PwAHA. Apart from FVIII, no other coagulation factor levels, such as FX, FIX or fibrinogen, could be determined owing to limited sample volumes. In addition, there were a small number of samples, and only 200 U/kg of rpFVIII was used for investigation of coagulation potential. Owing to the small sample size, no tests could be performed to statistically compare rpFVIII cross-reactivity between Japanese and Caucasian patients for equivalence. Nevertheless, our findings demonstrate that the cross-reactivity to rpFVIII in Japanese PwAHA is similar to that reported in Caucasian patients. In addition, our results suggest that an initial clinical dose of rpFVIII 200 U/kg can restore coagulation potentials to normal levels. Monitoring of FVIII:C after administration of rpFVIII may be more informative than measuring pFVIII inhibitor titer before administration of rpFVIII.

## Supplementary Information

Below is the link to the electronic supplementary material.

Supplementary file1 (PDF 402 KB)

## Data Availability

The data supporting the in vitro results presented in this article are available from the corresponding author on reasonable request.
