# Supplementary material for: The in vitro cross-reactivity and blood coagulation potential of recombinant porcine factor VIII in Japanese patients with acquired hemophilia A
Source: Int J Hematol. 2024 Oct 30;121(1):45–55. doi: 10.1007/s12185-024-03854-5 (PMC11742282; doi:10.1007/s12185-024-03854-5)
Supplement: Supplementary file 1 — Supplementary file1 (PDF 402 KB) [file 12185_2024_3854_MOESM1_ESM.pdf]

SUPPLEMENTARY MATERIAL

SUPPLEMENTARY TABLE 1 FVIII:C and CWA parameters in plasma from PwAHA

**Patient**	**FVIII:C (%)**		**CT (sec)**		**|min1|**		**|min2|**		**Ad|min1|**		**Ad|min2|**	
	**Pre[a]**	**Post[a]**	**Pre**	**Post**	**Pre**	**Post**	**Pre**	**Post**	**Pre**	**Post**	**Pre**	**Post**
**1**	< 1.0	398	83.9	30.1	6.407	10.105	0.788	1.594	6.706	10.798	0.703	1.703
**2**	< 1.0	296	120	43.1	0.885	5.070	0.022	0.605	1.020	6.899	0.025	0.824
**3**	7.5	186	83.9	37.4	2.344	7.331	0.106	1.027	2.876	8.923	0.131	1.249
**4[b]**	< 1.0	217	–	–	–	–	–	–	–	–	–	–
**5**	33	179	114	26.9	0.600	3.491	0.028	0.561	1.110	10.742	0.051	1.725
**6**	5.7	299	121	26.9	0.919	5.843	0.035	0.951	1.166	10.706	0.045	1.742
**7**	2.1	412	72.6	31.6	2.0845	6.168	0.138	0.945	2.666	9.645	0.176	1.478
**8**	1.1	116	143	33.6	0.365	3.797	0.028	0.596	0.695	10.069	0.053	1.579
**9**	< 1.0	291	84.8	28.1	1.089	3.448	0.050	0.574	2.287	11.451	0.105	1.906
**10**	12	455	69.6	25.5	2.357	5.562	0.133	0.919	3.529	10.935	0.199	1.808
**11**	< 1.0	480	75.2	24.4	0.922	2.433	0.050	0.421	2.897	11.955	0.155	2.066
**12**	< 1.0	573	114	32.5	0.864	6.010	0.024	0.888	1.045	9.162	0.029	1.354
**13**	2.1	476	71.4	24.3	1.826	5.334	0.085	0.886	2.712	10.829	0.126	1.798
**14**	1.4	229	97.1	35.8	1.502	6.831	0.076	1.017	1.751	9.635	0.088	1.435
**15**	< 1.0	355	103	26.5	1.035	6.233	0.057	1.024	1.307	11.399	0.072	1.873
**16**	< 1.0	484	112	31.8	1.278	4.591	0.050	0.730	1.876	9.739	0.073	1.547
**Mean (SD)**	4.1 (8.4)	340 (134)	97.6 (22.3)	30.6 (5.3)	1.632 (1.457)	5.483 (1.876)	0.111 (0.191)	0.849 (0.287)	2.243 (1.508)	10.192 (1.264)	0.135 (0.166)	1.606 (0.310)

Ad|min1| adjusted |min1|, *Ad|min2|* adjusted |min2|, *CT* clotting time, *CWA* clot waveform analysis, *FVIII:C* factor VIII activity, *|min1|* maximum coagulation velocity, *|min2|* maximum coagulation acceleration, *PwAHA* patients with acquired hemophilia A

[a]Pre = before addition of 5 U/mL rpFVIII (dose equivalent to 200 U/kg), post = after addition of 5 U/mL rpFVIII

[b]Measurements could not be performed for one patient owing to insufficient volume of stored plasma

SUPPLEMENTARY TABLE 2 TGA parameters in plasma from PwAHA.

Patient	FVIII:C (%)		Lag time (min)		ETP (nM*min)		Peak thrombin (nM)		Time to peak (min)	
	Pre[a]	Post[a]	Pre	Post	Pre	Post	Pre	Post	Pre	Post
**1**	< 1.0	398	10.0	8.5	2668	3930	141	503	26.4	11.7
**2**	< 1.0	296	6.5	6.2	3551	3589	173	285	20.3	13.2
**3**	7.5	186	11.4	9.9	2668	3833	106	350	28.1	15.0
**4[b]**	< 1.0	217	–	–	–	–	–	–	–	–
**5**	33	179	8.0	6.5	1609	2104	59.9	80.7	18.8	18.6
**6**	5.7	299	8.8	9.7	1904	2546	72.3	187	22.9	17.1
**7**	2.1	412	8.4	6.3	2205	3096	75.3	178	18.6	16.1
**8**	1.1	116	8.8	8.7	1278	2619	52.6	176	20.7	16.1
**9**	< 1.0	291	10.2	7.7	2827	3143	54.1	160	20.4	17.7
**10**	12	455	9.0	7.9	2575	2994	69.1	283	22.1	12.0
**11**	< 1.0	480	6.0	6.1	1967	2801	90.0	231	12.8	11.8
**12**	< 1.0	573	7.7	8.4	2305	3249	76.7	185	22.5	17.1
**13**	2.1	476	5.4	6.4	2182	3255	97.0	293	17.6	11.4
**14**	1.4	229	9.9	7.4	2556	2495	112	158	18.6	16.0
**15**	< 1.0	355	5.8	5.1	2289	2492	122	332	16.1	8.6
**16**	< 1.0	484	5.3	5.3	2575	3105	144	254	16.5	10.9
**Mean (SD)**	4.1 (8.4)	340 (134)	8.1 (1.9)	7.3 (1.5)	2344 (542)	3016 (519)	96.2 (36.1)	244 (103)	20.2 (3.9)	14.2 (3.0)

FVIII:C factor VIII activity, *ETP* endogenous thrombin potential, *PwAHA* patients with acquired hemophilia A, *rpFVIII* recombinant porcine factor VIII, *TGA* thrombin generation assay

[a]Pre = before addition of 5 U/mL rpFVIII (dose equivalent to 200 U/kg), post = after addition of 5 U/mL rpFVIII

[b]Measurements could not be performed for one patient owing to insufficient volume of stored plasma

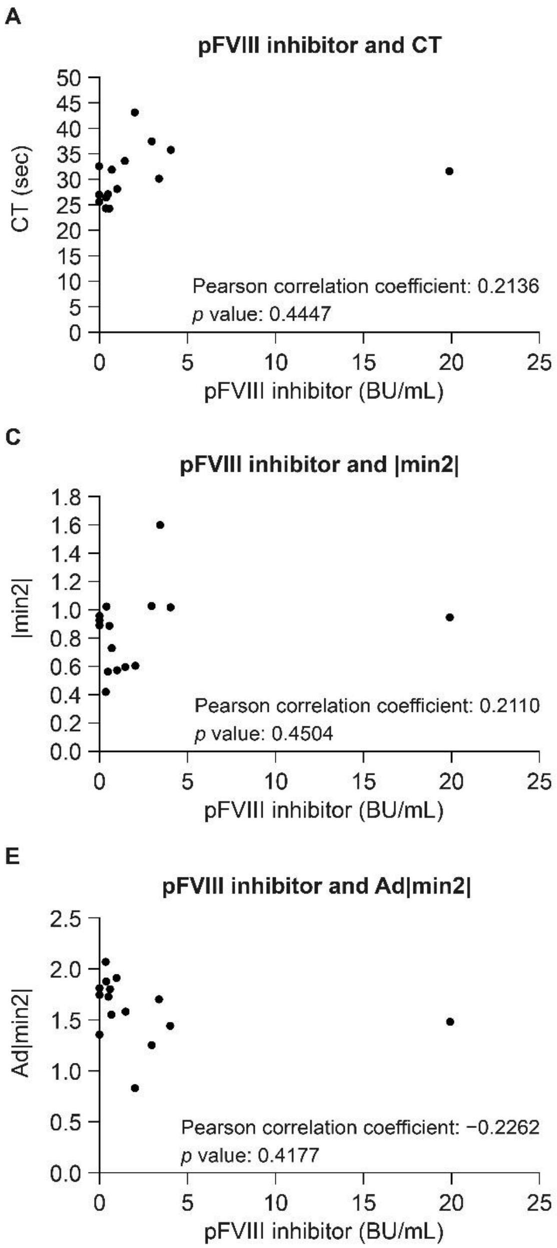

SUPPLEMENTARY FIG. 1 Correlation between pFVIII inhibition titer and CWA parameters in plasma from PwAHA. (A) CT, (B) |min1|, (C) |min2|, (D) Ad|min1| and (E) Ad|min2| were compared to the porcine inhibition titer determined for each sample by the Bethesda assay. N = 15 for all comparisons

Ad|min1| adjusted |min1|, *Ad|min2|* adjusted |min2|, *BU* Bethesda units, *CT* clotting time, *CWA* clot waveform analysis, *|min1|* maximum coagulation velocity, *|min2|* maximum coagulation acceleration, *pFVIII* porcine factor VIII, *PwAHA* patients with acquired hemophilia A

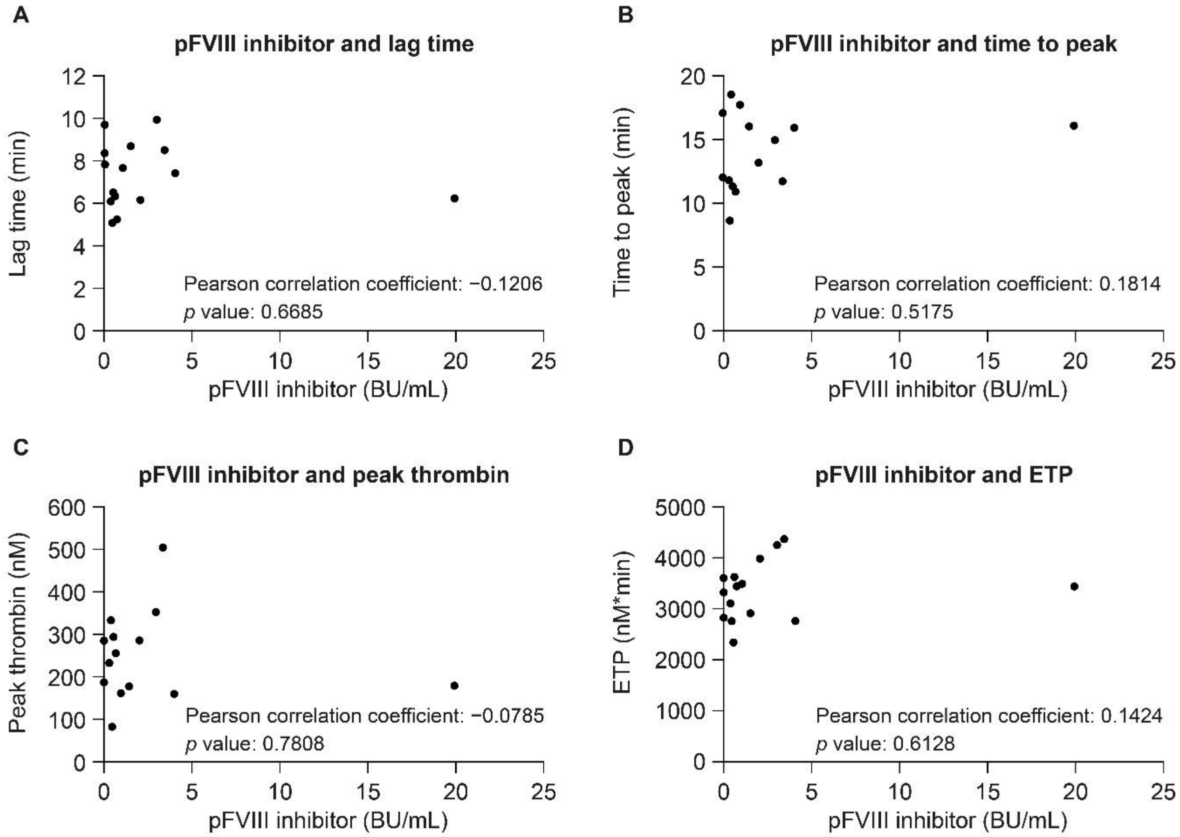

SUPPLEMENTARY FIG. 2 Correlation between pFVIII inhibition titer and thrombin generation assay parameters in plasma from PwAHA. (A) lag time, (B) time to peak thrombin, (C) peak thrombin and (D) ETP parameters were compared to the porcine inhibition titer determined for each plasma sample by the Bethesda assay. N = 15 for all comparisons

BU Bethesda units, *ETP* endogenous thrombin potential, *pFVIII* porcine factor VIII, *PwAHA* patients with acquired hemophilia A
